# Supplementary material for: An original infection model identifies host lipoprotein import as a route for blood-brain barrier crossing
Source: Nat Commun. 2020 Nov 30;11:6106. doi: 10.1038/s41467-020-19826-2 (PMC7704634; doi:10.1038/s41467-020-19826-2)
Supplement: Supplementary file 1 — Supplementary Information [file 41467_2020_19826_MOESM1_ESM.pdf]

Supplementary figure 1

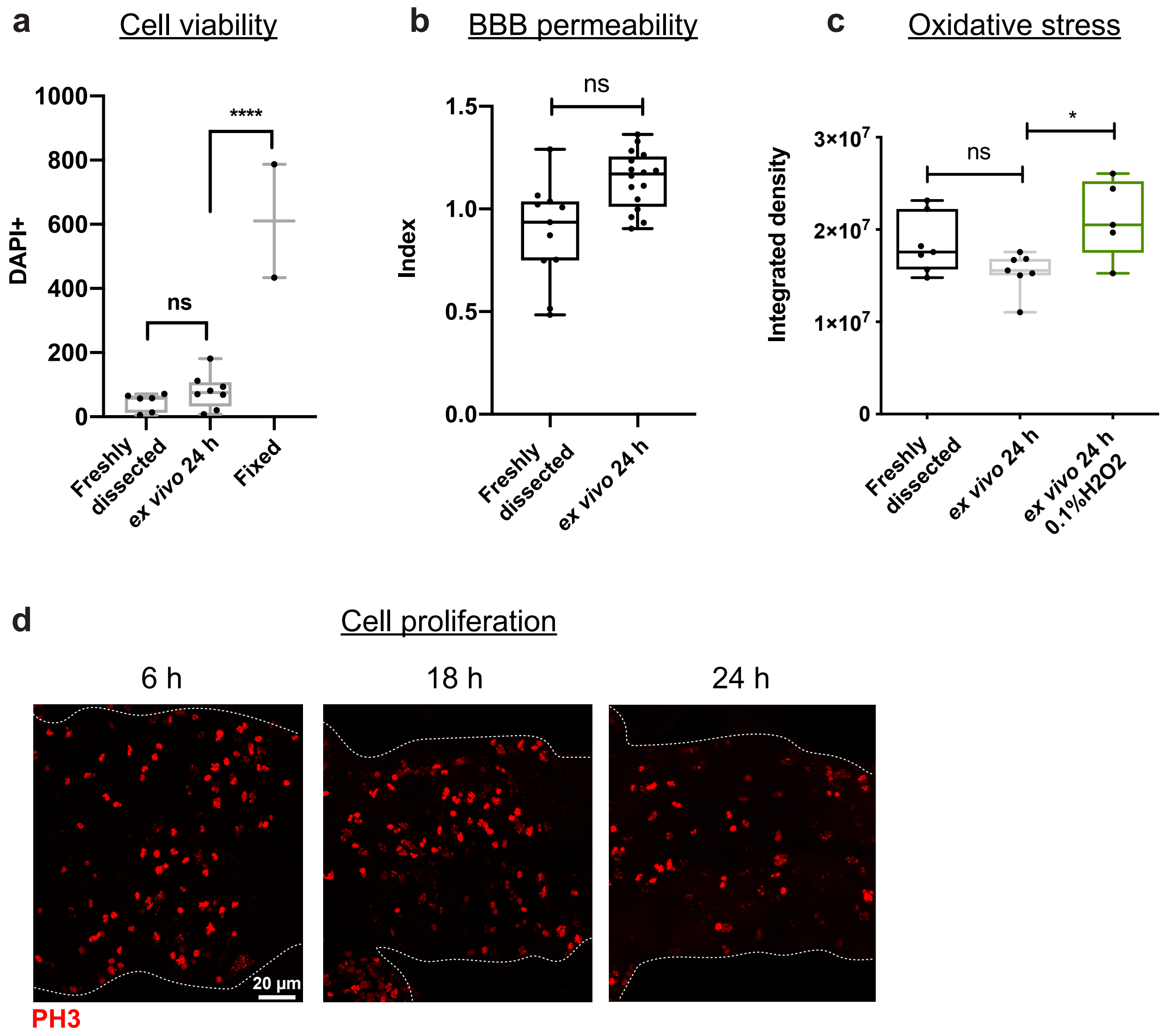

Supplementary Figure 1: Drosophila as a platform to screen for mammalian neuro-invasive pathogens. Related to Figure 1.

a. Cell death monitored by DAPI penetration in freshly dissected (n = 6), after 24 h of *ex vivo* culture (n = 8), and fixed (n = 2) brains. Cell viability is not affected in *ex vivo* compared to freshly dissected brains. Two-tailed Mann-Whitney test: p(fresh vs *ex vivo*) = 0.1079.

b. BBB permeability after 24 h *ex vivo* culture showed a small, although non-significant, increase. One-way ANOVA test followed by Tukey's multiple comparisons test generated adjusted p-values: freshly dissected (n = 11) vs *ex vivo* (n = 16) ns, p = 0.0787.

c. Oxidative stress monitored through DHE (Dihydroethidium) staining in brains freshly dissected (n = 7), after 24h of *ex vivo* culture (n = 7), or exposed to 0.1% (n = 5) H_2O_2. One-way ANOVA test followed by Tukey's multiple comparisons test generated adjusted p-values: p(fresh vs *ex vivo*) = 0.2096, p(*ex vivo* vs *ex vivo*+0.1%H_2O_2) = 0.0172.

d. Representative confocal images of Drosophila larval brains stained for phospho-histone 3 (PH3, red) after 6, 18, and 24 h of *ex vivo* culture.

For results presented as box and whisker plots: whiskers mark the minimum and maximum, the box includes the 25th–75th percentile, and the line in the box is the median.

*, $p \leq 0.05$; ****, $p \leq 0.0001$; ns, not significant.

Source data are provided as a Source Data file for a-c.

Supplementary figure 2

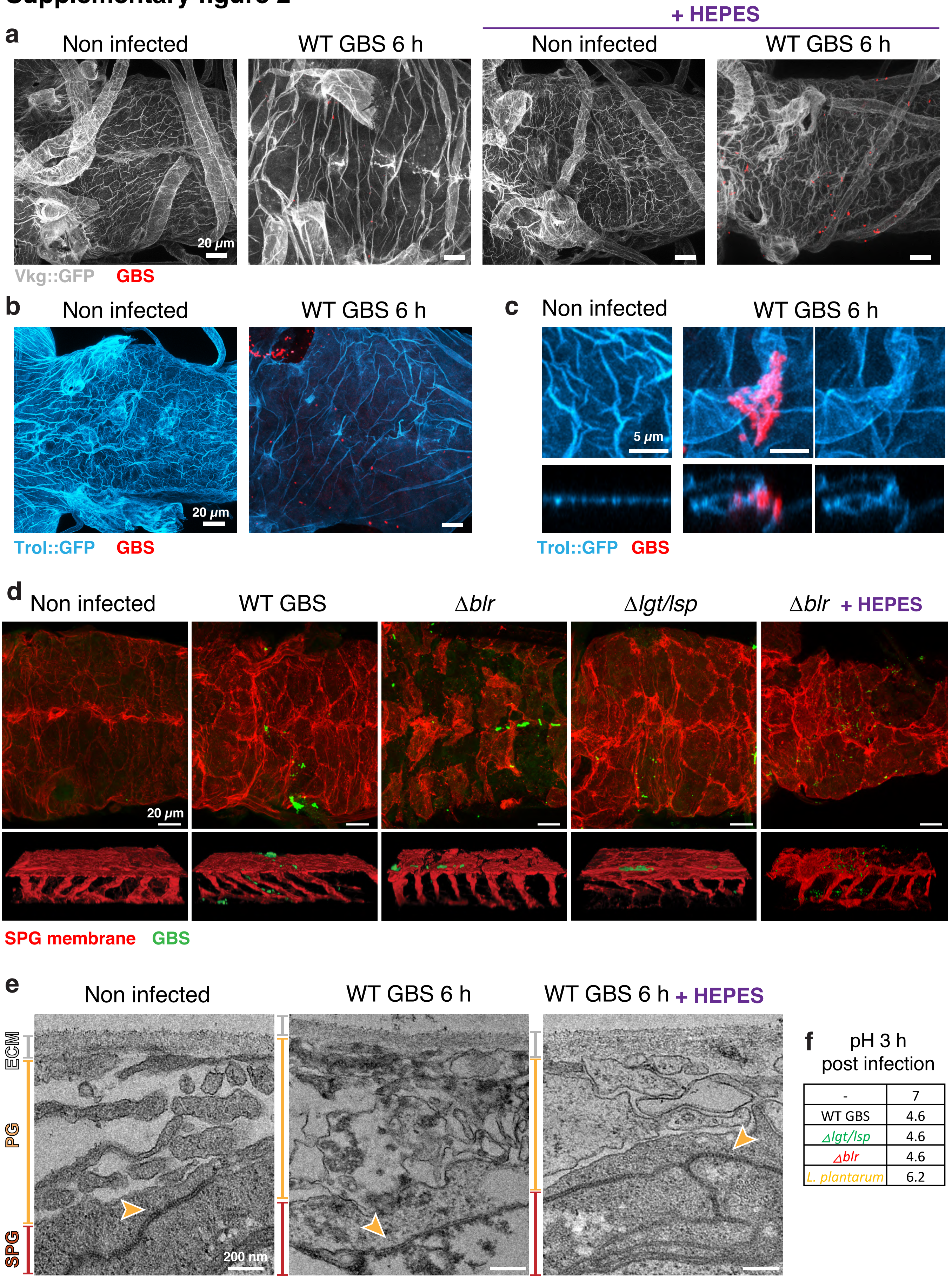

-	7
WT GBS	4.6
Δlgt/lsp	4.6
Δblr	4.6
L. plantarum	6.2

Supplementary Figure 2: GBS uses a variety of mechanism to cross cellular layers. Related to Figure 2.

a. Confocal images of the ECM layer (top view) labelled for Collagen IV (*vkg::GFP*) for non-infected brain and brain infected with WT GBS at 6 h post infection, with and without acidosis.

b. Confocal images of the ECM layer (top view) labelled for Perlecan (*trol::GFP*) for non-infected brain and brain infected with WT GBS at 6 h post infection.

c. Close-up of confocal images (top view and orthogonal view) of non-infected and brain infected with WT GBS at 6 h post infection, showing Perlecan staining (*trol::GFP*, green) and GBS (red).

d. Confocal images of the Drosophila BBB (top view and 3D orthogonal view) labelled for the SPG membrane in red (*mdr65-mtd-tomato*) at 6 h post-infection with WT, *Δblr* and *Δlgt/lsp* GBS strains (all green). Higher alteration of SPG labelling was observed under infection by *Δblr* GBS compared to infection by WT and *Δlgt/lsp* GBS. Such alteration was partially rescued by buffering medium acidity.

e Transmission electron microscopy (TEM) pictures of a non-infected brain and brains infected by GBS 6 h post infection with or without acidosis, showing the different BBB layers and septate junctions (orange arrow).

f. pH measurements of culture media non-inoculated and inoculated with WT, *Δlgt/lsp,* and *Δblr* GBS and *L. plantarum* for 3 h.

Supplementary figure 3

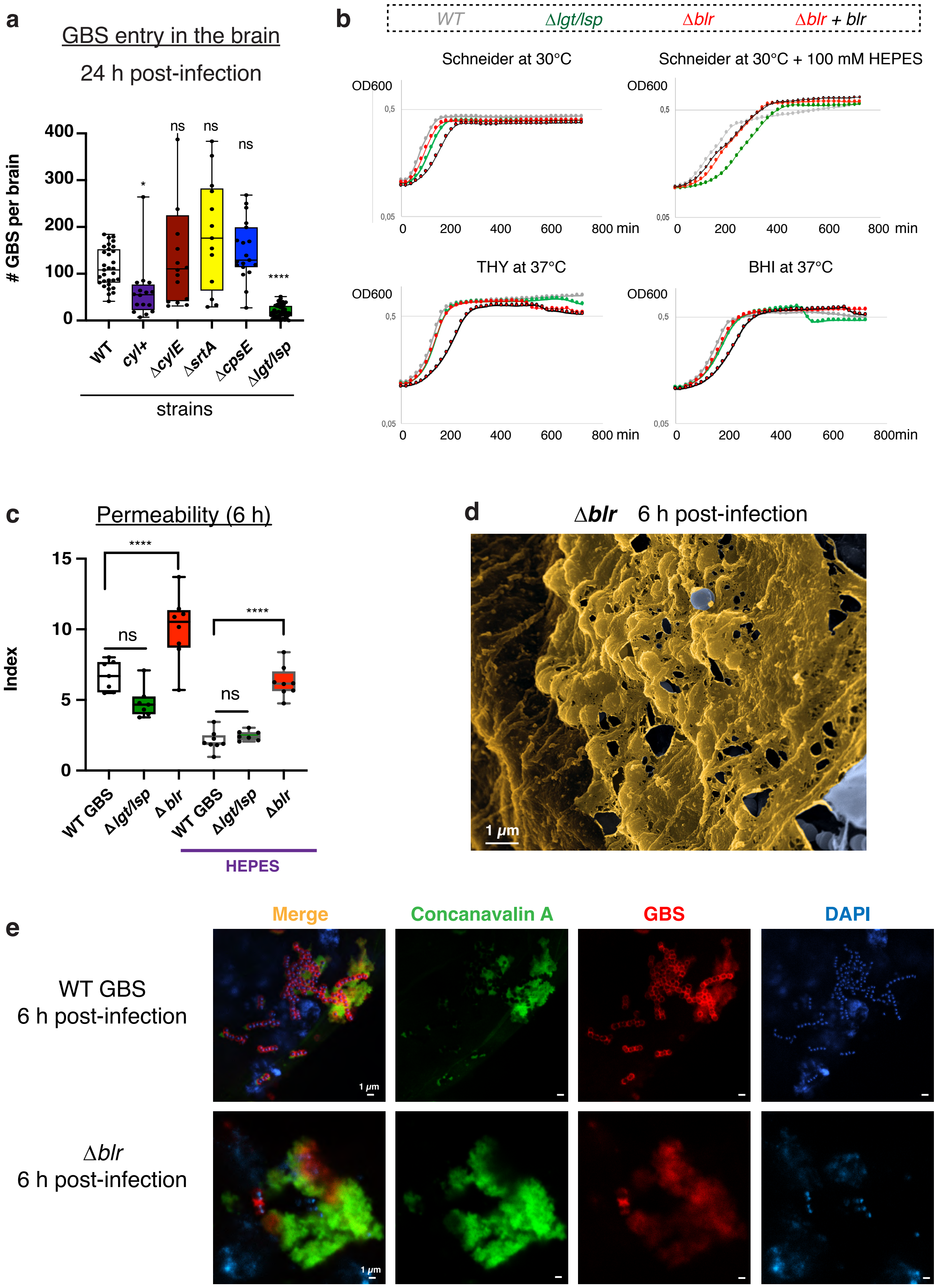

Supplementary Figure 3: Roles of selected GBS factors on pathogenic invasion, BBB parameters and bacterial fitness. Related to Figure 3.

a. Screening of selected GBS virulence factors and surface molecules at 24 h post infection identified GBS lipoproteins (*Δlgt/lsp* mutant*)* as a crucial factor for GBS brain invasion. A Kruskal-Wallis test followed by Dunn's multiple comparisons test generated adjusted p-values between WT GBS (n = 31) and: *cyl+* (n = 16), p = 0.0239; *ΔcylE* (n = 16), p > 0.9999; *ΔSrtA* (n = 13), p > 0.9999; *ΔcpsE* (n = 19), p > 0.9999; *Δlgt/lsp* (n = 43), $p < 10^{-10}$.

b. Growth curves of WT, *Δlgt/lsp, Δblr*, and *Δblr+blr* GBS strains at 30 °C in Drosophila Schneider's medium complemented or not with HEPES, and at 37 °C in THY and BHI media.

c. BBB permeability tests for brains infected by the different GBS strains (6 h post-infection) with and without HEPES. In both cases, BBB permeability is higher in brains infected with GBS-*Δblr* compared to other strains. One-way ANOVA tests followed by Tukey's multiple comparisons test generated adjusted p-values. Without HEPES: p(WT GBS vs *Δlgt/lsp*) = 0.1036; p(WT vs *Δblr*) = 0.0002. WT GBS (n = 7); *Δlgt/lsp* GBS (n = 7) and *Δblr* GBS (n = 8). With HEPES: p(WT vs *Δlgt/lsp*) = 0.9923; p(WT vs *Δblr*) = $1.89 * 10^{-6}$. WT GBS (n = 8), *Δlgt/lsp* GBS (n = 7) and *Δblr* GBS (n = 8). Moreover, p(*Δblr* vs *Δblr*+HEPES) = $1.29 * 10^{-5}$.

d. SEM picture of a brain infected by *Δblr* GBS 6 h post-infection showing a biofilm-like matrix, colorised in yellow. This picture represents a rare event which has been observed on 2 brains out of 5 over 2 experiments (40%).

e. Close-up of confocal picture (top view) of brains infected by WT GBS and *Δblr* at 6 h post-infection and stained for GBS (red), DNA (DAPI, blue) and biofilm polysaccharides (Concanavalin A, green). Concavalin A staining is seen on top and between bacteria. Both living bacteria (identified by the DAPI staining of their DNA) and dead bacteria can be found in biofilms. This picture represents a rare event which has been observed on 1 to 2 brains out of 7 brains per condition (15%).

For results presented as box and whisker plots: whiskers mark the minimum and maximum, the box includes the 25th–75th percentile, and the line in the box is the median.

*, p ≤ 0.05; ****, p ≤ 0.0001; ns, not significant.

Source data are provided as a Source Data file for a and c.

Supplementary figure 4

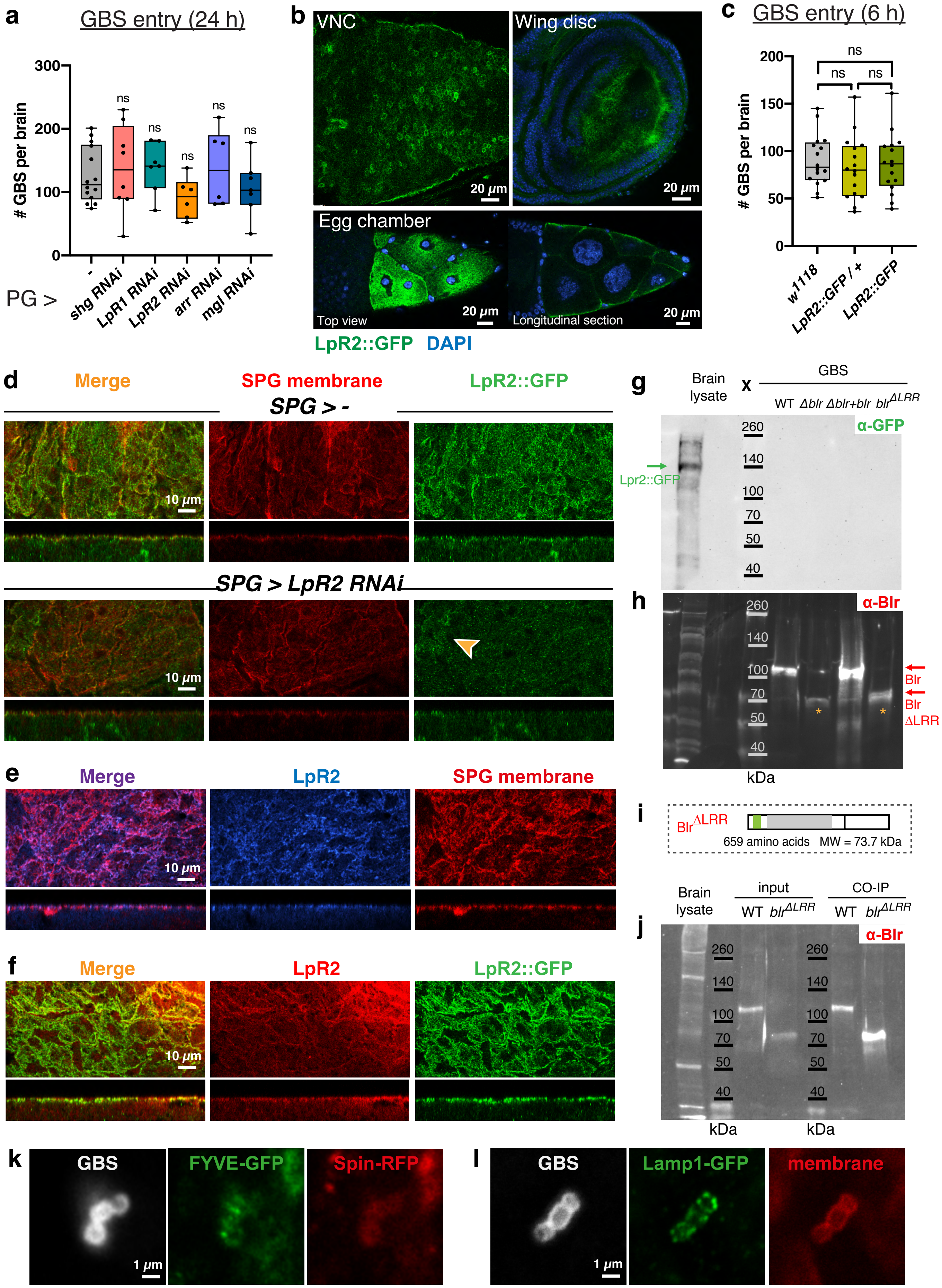

Supplementary Figure 4: The endocytic receptor LpR2 mediates specific passage of the SPG by GBS. Related to Figure 4.

a. GBS brain entry is not affected, 24 h post-infection, after knocking down E-cadherin (*shg*) and lipoprotein receptors in the PG of Drosophila. One-way ANOVA test followed by Dunnett's multiple comparisons test generated adjusted p-values: - (n = 14); *shg*, p = 0.9885 (n = 8); *LpR1*, p = 0.9858 (n = 7); *LpR2*, p = 0.4932 (n = 6); *arr*, p = 0.9886 (n = 6); *mgl*, p = 0.8845 (n = 7).
b. Confocal images showing LpR2::GFP localisation in larval VNC, wing disc (pouch) as well as in adult egg chamber. In the VNC, LpR2::GFP was detected in neurons in addition to its expression in the BBB. DAPI, blue.
c. WT GBS entry at 6 h post infection is similar between control genotype (w^{1118} background), *LpR2::GFP* knock-in heterozygous and *LpR2::GFP* knock-in heterozygous (n = 16 for each of the three conditions). One-way ANOVA followed by Tukey's multiple comparisons test generated adjusted p-values: p(w1118 vs *LpR2::GFP/+*) = 0.7335. p(w1118 vs *LpR2::GFP*) = 0.9315. p(*LpR2::GFP/+* vs *LpR2::GFP*) = 0.9182.
d. Confocal images of top close-up view (top panels) and orthogonal view (bottom panels) showing LpR2::GFP signal and colocalisation with the SPG membrane (*mdr65-GAL4 > mCD8-RFP*, red) in a control brain (*SPG* > -) and in a brain in which *LpR2* has been specifically knocked-down in the SPG (*SPG > LpR2-RNAi*). LpR2::GFP signal is strongly decreased upon *LpR2* RNAi, although some can still be detected (orange arrowheads).
e. Confocal images of top close-up view (top panels) and orthogonal view (bottom panels) showing LpR2 (anti-LpR2, blue) and SPG (*mdr65-mtd-tomato,* red) colocalisation in the brain of a third instar larva.
f. Confocal images of top close-up view (top panels) and orthogonal view (bottom panels) showing LpR2 (anti-LpR2, blue) and LpR2::GFP colocalisation in the brain of a third instar larva.
g. Western-Blot showing brain lysate and bacterial lysate inputs detected by an anti-GFP antibody. A signal is only detected in the lane corresponding to the brain lysate. A major band around 140 kDa corresponds to LpR2::GFP.
h. Western-Blot showing brain lysate and bacterial lysate inputs detected by an anti-Blr antibody. WT Blr is detected as a band around 100 kDa, et Blr without the LRR domains (659 amino acids and MW = 73.73 kDA) is detected as a band around 75 kDa. A non-specific band just below $Blr^{\Delta LRR}$ is also detected in all samples (orange star). The (*Δblr + blr*) condition results in Blr overexpression.
i. Schematic structure of $Blr^{\Delta LRR}$.
j. Co-immunoprecipitation experiment between LpR2::GFP immobilised on beads and bacterial lysates of WT GBS and $blr^{\Delta LRR}$ GBS, detected with an antibody against Blr. Lpr2 is still able to interact with Blr depleted of the LRR domain.
k - l. Colocalisation of GBS (white) with (k) a marker for lysosome (Lamp1-GFP) in green within the SPG membrane (*mdr65-mtd-Tomato*, red) and (l) both endosomal and lysosomal markers (FYVE-GFP and Spin-RFP, respectively in green and red), pinpointing GBS presence in autophagosomes.
For results presented as box and whisker plots: whiskers mark the minimum and maximum, the box includes the 25th–75th percentile, and the line in the box is the median.
ns, not significant.
Source data are provided as a Source Data file for a, c, g, h and j.

Supplementary figure 5

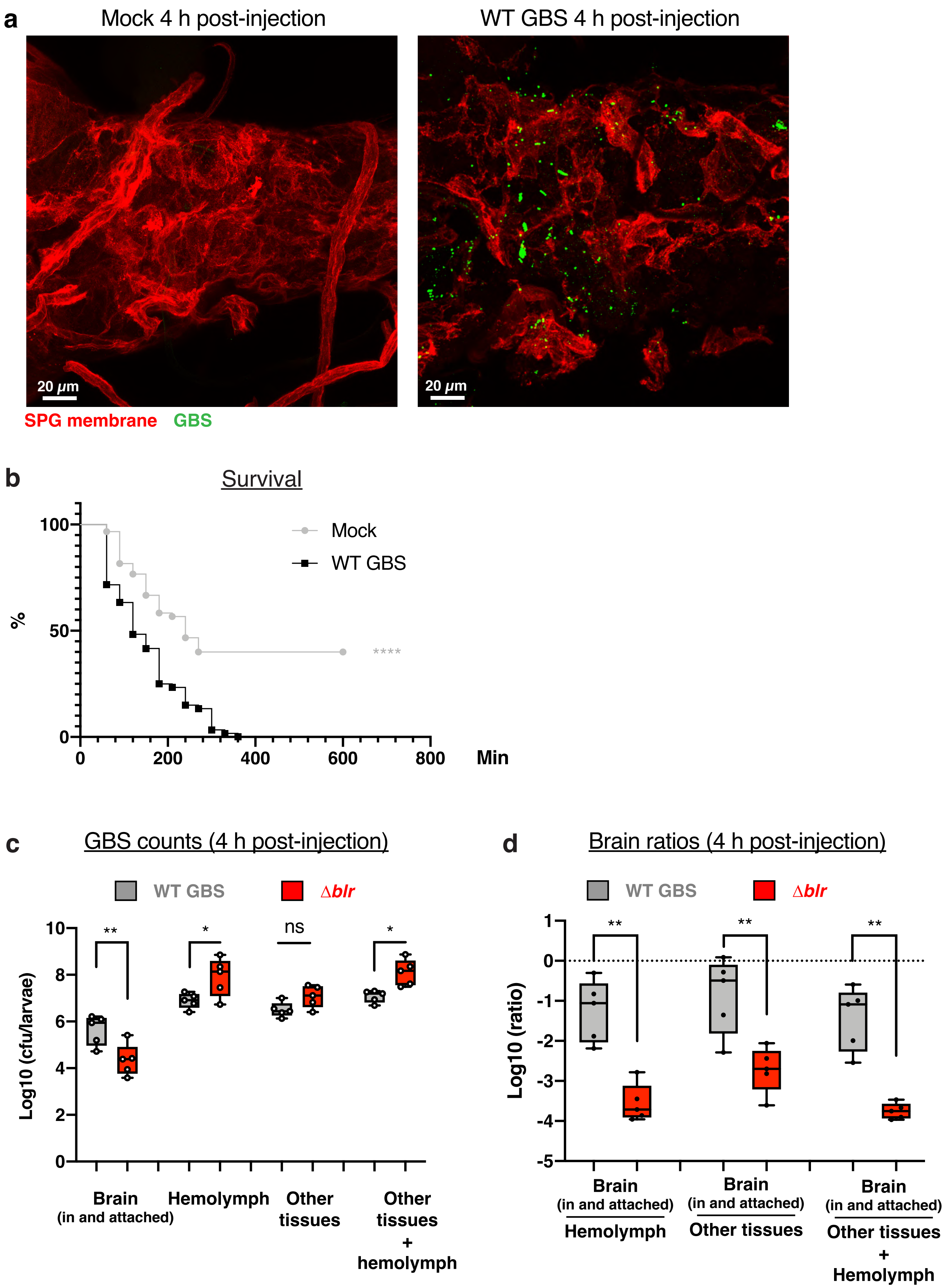

Supplementary Figure 5: GBS brain entry *in vivo* through pathogen microinjection into the larval hemolymph. Related to Figure 5.

a. Confocal image of the SPG membrane (top view, red) at 4 h post-injection with mock and WT GBS (green) *in vivo*. A strong alteration of SPG labelling was observed, a rare event which has been observed on 2 out of 13 brains over 3 experiments.

b. Kaplan-Meier survival curves for larvae injected with mock and WT GBS (n = 60 for each condition) followed up to 10 h after injection show that all infected larvae die between 4 h and 5 h post-injection. Mock-injected animals can go into metamorphosis and survive up to adulthood. Log rank test: p(WT GBS vs mock) = $8.77 * 10^{-8}$.

c. Cfu counts in and attached to the brain, in the hemolymph and in other tissues at 4 h after injection in the hemolymph of WT larvae of WT GBS (n = 5) and *Δblr* GBS (n = 5). Two-tailed unpaired Student's t test for each compartment: p(brain) = 0.0157, p(hemolymph) = 0.0341, p(other tissues) = 0.0562, p(other tissues+hemolymph) = 0.0063.

d. Ratio (in Log10) of bacterial counts in and attached the brain versus hemolymph, brain versus other tissues, and brain versus other tissues+hemolymph at 4 h post injection.

Two-tailed unpaired Student's t test for each compartment: p(brain/hemolymph) = 0.0010, p(brain/other tissues) = 0.0064, p(brain/other tissues+hemolymph) = 0.0011.

For results presented as box and whisker plots: whiskers mark the minimum and maximum, the box includes the 25th–75th percentile, and the line in the box is the median.

*, $p \leq 0.05$; **, $p \leq 0.01$; ns, not significant.

Source data are provided as a Source Data file for b-d.

Supplementary figure 6

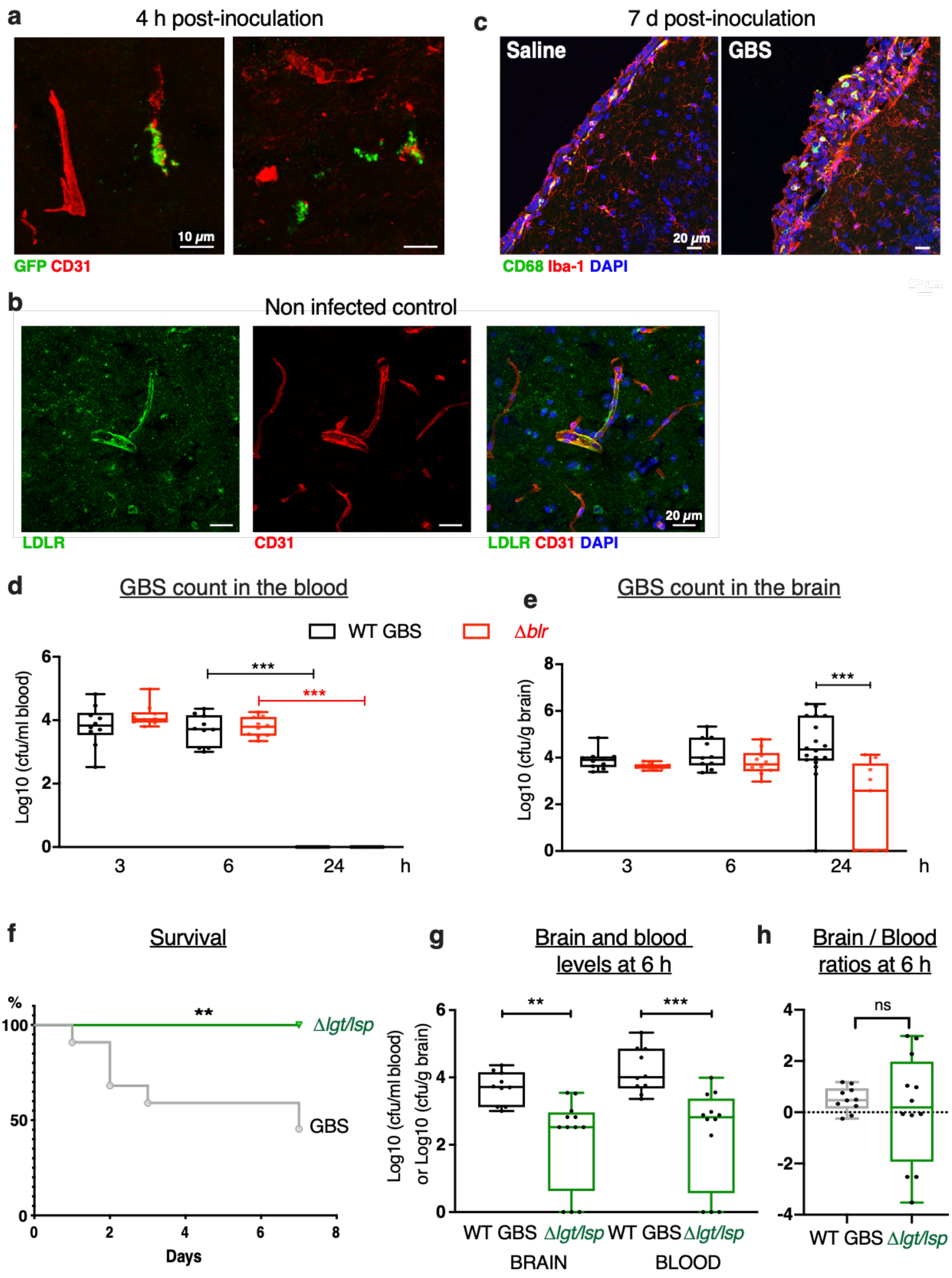

Supplementary Figure 6: GBS infection in mice. Related to Figure 6.
a. Confocal images showing GBS (green) around destroyed brain capillaries (CD31, red) at 4 h post-infection.
b. Confocal image of a non-infected control mouse sagittal brain section displaying colocalisation of LDLR (green) and CD31 (endothelial cell marker; red) on brain capillaries.
c. Confocal images of sagittal brain sections of mice injected with saline or GBS WT at 7 d after inoculation, immunostained against Cluster of Differentiation 68 (CD68; green) and ionized calcium-binding adapter molecule 1 (Iba-1; red). Meningitis hallmarks including meningeal thickening and leukocyte accumulation in the meninges are apparent in the case of GBS WT-inoculated mouse as compared with the saline-injected control.
d. Bacterial levels in the blood [log10(cfu/ml)] for mice infected with WT and *Δblr* GBS at 3 (n = 10 for both), 6 (n = 10 for both), or 24 h (n = 18 and n = 17, respectively) after inoculation. Kruskal-Wallis followed by Dunn's multiple comparisons test: p(WT GBS 6 h vs 24 h) = 0.0003, p(Δ*blr* 6 h vs 24 h) = 0.0002.
e. Bacterial levels in the brain (including bacteria found in the parenchyma and inside the capillaries) [log10(cfu/g)] for GBS WT and GBS *Δblr* infected mice at 3 (n = 10 for both), 6 (n = 10 for both), or 24 h (n = 18 and n = 17, respectively) after inoculation. Kruskal-Wallis followed by Dunn's multiple comparisons test: p(24 h WT GBS vs Δ*blr*) = 0.0005.
f. Kaplan-Meier survival curves of mice infected with WT (n = 22) or Δ*lgt/lsp* (n = 10) GBS. Log-Rank test: p = 0.0055.
g. GBS levels in the brain [log10(cfu/g)] and blood [log10(cfu/mL)] at 6 h post-injection for mice infected with WT (n = 10) and *Δlgt/lsp* (n = 12) GBS. Student's t test with Welch's correction: p(brain) = 0.0009, and p(Blood) = 0.0023.
h. Brain/Blood ratio at 6 h post-injection. Two-tailed Student's t test*:* p = 0.6085.
For results presented as box and whisker plots: whiskers mark the minimum and maximum, the box includes the 25th–75th percentile, and the line in the box is the median.
**, $p \leq 0.01$; ***, $p \leq 0.001$; ns, not significant.
Source data are provided as a Source Data file for d-h.
