# Supplementary material for: An original infection model identifies host lipoprotein import as a route for blood-brain barrier crossing
Source: Nat Commun. 2020 Nov 30;11:6106. doi: 10.1038/s41467-020-19826-2 (PMC7704634; doi:10.1038/s41467-020-19826-2)
Supplement: Supplementary file 3 — Reporting Summary [file 41467_2020_19826_MOESM3_ESM.pdf]

nature research

Corresponding author(s): Pauline Spéder

Last updated by author(s): Oct 27, 2020

Reporting Summary

Nature Research wishes to improve the reproducibility of the work that we publish. This form provides structure for consistency and transparency in reporting. For further information on Nature Research policies, see our Editorial Policies and the Editorial Policy Checklist.

Statistics

For all statistical analyses, confirm that the following items are present in the figure legend, table legend, main text, or Methods section.

n/a	Confirmed	
☐	☒	The exact sample size (*n*) for each experimental group/condition, given as a discrete number and unit of measurement
☐	☒	A statement on whether measurements were taken from distinct samples or whether the same sample was measured repeatedly
☐	☒	The statistical test(s) used AND whether they are one- or two-sided<br>*Only common tests should be described solely by name; describe more complex techniques in the Methods section.*
☒	☐	A description of all covariates tested
☐	☒	A description of any assumptions or corrections, such as tests of normality and adjustment for multiple comparisons
☐	☒	A full description of the statistical parameters including central tendency (e.g. means) or other basic estimates (e.g. regression coefficient) AND variation (e.g. standard deviation) or associated estimates of uncertainty (e.g. confidence intervals)
☐	☒	For null hypothesis testing, the test statistic (e.g. *F*, *t*, *r*) with confidence intervals, effect sizes, degrees of freedom and *P* value noted<br>*Give P values as exact values whenever suitable.*
☒	☐	For Bayesian analysis, information on the choice of priors and Markov chain Monte Carlo settings
☒	☐	For hierarchical and complex designs, identification of the appropriate level for tests and full reporting of outcomes
☒	☐	Estimates of effect sizes (e.g. Cohen's *d*, Pearson's *r*), indicating how they were calculated

Our web collection on statistics for biologists contains articles on many of the points above.

Software and code

Policy information about availability of computer code

Data collection	Zeiss Zen software (2012 S4) was used for confocal acquisition of Drosophila images.<br>Image Lab Software (2020 6.1) was used to acquire western blot images.<br>Leica Application Suite X software version 3.5.5 was used for confocal acquisition of mouse images.<br>TIA software V4 was used for Transmission Electron Microscopy with a TECNAI SPIRIT microscope.<br>JEOL software module (PC-SEM Main Executable version 3.31.13) was used for Scanning Electron Microscopy.<br>Gen5 data analysis software (v.3.03) was used to record absorbance measurements on a Biotek Synergy 2 microplate.
Data analysis	ImageJ (2020 2.1.0/1,53c), Icy (2.0.3.0) and Volocity (6.3) were used to analyse Drosophila confocal data.<br>ImageJ 1.52p was used for image processing of mouse confocal images.<br>GraphPad Prism software was used for all Drosophila (2020 8.4.2 (464) and mouse (version 7) statistical analysis.<br>GPower version 3.1. was used to estimate sample size for mouse infection.

For manuscripts utilizing custom algorithms or software that are central to the research but not yet described in published literature, software must be made available to editors and reviewers. We strongly encourage code deposition in a community repository (e.g. GitHub). See the Nature Research guidelines for submitting code & software for further information.

Data

Policy information about availability of data

All manuscripts must include a data availability statement. This statement should provide the following information, where applicable:

- Accession codes, unique identifiers, or web links for publicly available datasets
- A list of figures that have associated raw data
- A description of any restrictions on data availability

The datasets generated during and/or analysed during the current study are available from the corresponding author on reasonable request.
Source data are available with the paper for Fig. 4e and Supplementary Fig. 4g-h, j.

Field-specific reporting

Please select the one below that is the best fit for your research. If you are not sure, read the appropriate sections before making your selection.

- [x] Life sciences
- [ ] Behavioural & social sciences
- [ ] Ecological, evolutionary & environmental sciences

For a reference copy of the document with all sections, see nature.com/documents/nr-reporting-summary-flat.pdf

Life sciences study design

All studies must disclose on these points even when the disclosure is negative.

Sample size	For Drosophila experiments, no sample-size calculations were performed a priori. Sample sizes were determined by the number of processed samples (6-8 precisely staged dissected brains per experiment) and the number of independent experiments (mainly 2 to 3). This is routinely what we use in the lab for experimental design, with low variability between replicates observed from many experiments over the years (being partly due to the robustness of Drosophila genetics).<br><br>For mouse experiments sample sizes were determined a priori using GPower v. 3.1.
Data exclusions	No data have been excluded.
Replication	For Drosophila, all replications for bacterial counts, survival curves, permeability index, western-blotting and immunochemistry were successful. Most experiments have been performed at least 2 times, expect for the co-immunoprecipitation of BlrdLRR, which has been performed only once. The only difficult was for anti-LpR2 staining, which is variable. We however were able to observe a SPG localisation similar to LpR2::GFP in 13 out of 30 brains over 5 experiments (localisation depicted in Supplementary Figure 4e-f).<br>For mice, all experiments have been performed once. The Protocols Evaluation Committee that gives licenses for mouse experiments did not allow for independent experiments due to ethical issues and the principles of the 3 Rs (reduce, refine, replace).
Randomization	For Drosophila experiments, randomisation of larvae into groups was not possible most of the times as we were dealing with different genotypes that could not be mixed without loosing the ability to distinguish them easily (for exemple different RNAi lines driven by the same GAL4 line). For experiments using larvae of only one genotype (for exemple w1118 line for Figure 5c), larvae were randomly grouped in the different infection conditions.<br><br>For mice experiments, mice were randomly grouped in the experimental cohorts.
Blinding	Experimentators were not blinded, as most experiments either produced a visible, distinguishing phenotype (infected versus non-infected for exemple) or required to prepare and keep separated different infection conditions or different genotypes. However subsequent analysis by co-investigators was unbiased. For example, images were shown to another investigator without labelling the condition.

Reporting for specific materials, systems and methods

We require information from authors about some types of materials, experimental systems and methods used in many studies. Here, indicate whether each material, system or method listed is relevant to your study. If you are not sure if a list item applies to your research, read the appropriate section before selecting a response.

Materials & experimental systems

n/a	Involved in the study	
☐	☒	Antibodies
☒	☐	Eukaryotic cell lines
☒	☐	Palaeontology and archaeology
☐	☒	Animals and other organisms
☒	☐	Human research participants
☒	☐	Clinical data
☒	☐	Dual use research of concern

Methods

n/a	Involved in the study	
☒	☐	ChIP-seq
☒	☐	Flow cytometry
☒	☐	MRI-based neuroimaging

Antibodies

Antibodies used

1. PRIMARY
Rabbit anti-GBS (homemade), Mouse anti-S.pneumoniae (homemade), Rabbit anti-L.monocytogenes (R12, gift from M. Lecuit), Rabbit anti-L.innocua (R6, gift from M. Lecuit) (Dramsi et al., 1998 or Moura et al., 2019), Rabbit anti-Blr (Waldemarsson et al., 2006), Chicken anti-GFP (Abcam, ab13970, lot:GR3190550-10), Guinea Pig anti-LpR2 (gift from J. Culi, Parra-Peralbo and Culi., 2011), Rat anti-GFP (1/1000, Chromotek [3H9], lot:80626001AB), Biotinylated-Concavalin A (1/200, B-1005, Vector Laboratories), Rat anti-Cluster of Differentiation 68 (CD68; 1:100; Bio-Rad Antibodies, Oxford, UK; MCA1957GA), Rabbit polyclonal anti-ionized calcium-binding adapter molecule 1 (Iba-1; 1:400; FUJIFILM Wako Pure Chemical Corporation, Osaka, Japan; 019-19741), Rabbit anti-CD31 (1:50; Abcam, Cambridge, UK; ab28364), Goat anti-LDLR (1:100, R&D Systems, MN, USA; AF2255).
2. SECONDARY
Goat anti-Chicken IgY (H+L) Secondary Antibody, Alexa Fluor 488 ThermoFisher Scientific A11039 2079383
Goat anti-Rabbit IgG (H+L) Highly Cross-Adsorbed Secondary Antibody, Alexa Fluor 488 ThermoFisher Scientific A11034 1971418
Goat anti-Rabbit IgG (H+L) Highly Cross-Adsorbed Secondary Antibody, Alexa Fluor 546 ThermoFisher Scientific A11035 2129899
Goat anti-Rabbit IgG (H+L) Highly Cross-Adsorbed Secondary Antibody, Alexa Fluor 633 ThermoFisher Scientific A21071 1932492
Goat anti-Guinea Pig IgG (H+L) Highly Cross-Adsorbed Secondary Antibody, Alexa Fluor 633 ThermoFisher Scientific A21105 2045328
Goat anti-Guinea Pig IgG (H+L) Highly Cross-Adsorbed Secondary Antibody, Alexa Fluor 488 ThermoFisher Scientific A11073 1637243
Peroxidase AffiniPure Donkey Anti-Rat IgG (H+L) Jackson ImmunoResearch (712-035-153) 149509
Goat anti-Rat IgG (H+L) Cross-Adsorbed Secondary Antibody, Alexa Fluor 488 ThermoFisher Scientific A11006 (34745A)
Donkey anti-Goat IgG (H+L) Cross-Adsorbed Secondary Antibody, Alexa Fluor 488 ThermoFisher Scientific A11055 (1687906)
Goat anti-Rabbit IgG (H+L) Cross-Adsorbed Secondary Antibody, Alexa Fluor 546 ThermoFisher Scientific A11010 (1488578)
Donkey anti-Rabbit IgG (H+L) Highly Cross-Adsorbed Secondary Antibody, Alexa Fluor 546 ThermoFisher Scientific A10040 (2020130)

Validation

All commercial primary antibodies have been validated by suppliers as follows:
• chicken anti-GFP Abcam ab13970 (GR3190550-10). "The Abpromise guarantee covers the use of ab13970 for IHC-P, WB, ICC/IF, IHC-Fr, IHC-FoFr. The ab13970 antibody does cross-react with the many fluorescent proteins that are derived from the jellyfish Aequorea victoria. These are all proteins that differ from the original GFP by just a few point mutations (EGFP, YFP, mVenus, CFP, BFP etc.)."
• rat primary anti-GFP Chromotek [3H9] (80626001AB). Validated by Western-blot. A major band was observed at the correct molecular weight in the two positive(eGFP-transduced) cell lysates (293FT cells transduced with eGFP virus). No major bands were observed in the negativecontrol (untransduced cells 293FT cells). Some additional faint bands of lower molecular weight were also observed in the positive controls, this may be because the antibody was used at 1:500 instead of the recommended 1:1000 dilution. Microscope images of transduced cells are provided to demonstrate successful eGFP-transduction of cells.
• rabbit anti-phospho-Histone H3 (Ser10) Millipore 06-570 (3237504). Immunocytochemistry Analysis: Validation in HeLa and A431 cells. Immunoprecipitation Analysis: immunoprecipitated phospho Histone H3 (Ser10) from HeLa acid extract and Colcemid treated HeLa acid extracted RIPA lysate. Beadlyte® Assay Analysis: recognizes Histone H3 phosphorylated on Ser10 by Luminex assay.
• Biotinylated-Concavalin A Vector Laboratories (B-1005). "Detection of Glycoproteins using Lectins in Histochemistry, ELISA and WB applications."
• R&D Systems, AF2255 Goat polyclonal anti-LDLR UCH0117121. Detects mouse LDLR in direct ELISAs and Western blots. In direct ELISAs, approximately 10% cross-reactivity with recombinant human LDL R is observed.
• FUJIFILM Wako Chemicals, 019-19741 Rabbit polyclonal andi-Iba1 PTP5154. Reactive with human, mouse and rat Iba1. Purified by the antigen affinity chromatography from rabbit antisera and prepared in TBS solution. Contains no preservatives and stabilizers.
• Abcam, ab28364 Rabbit polyclonal andi-CD31 GR3247742-16. "Ab promise guarantee covers the use of ab28364 in IHC-P. IHC and ICC determine whether an antibody recognizes the correct protein based on cellular and subcellular localization. Antibody specificity is confirmed by looking at cells that either do or do not express the target protein within the same tissue. Initially, our scientists will review the available literature to determine the best cell lines and tissues to use for validation. We then check the protein expression by IHC/ICC to see if it has the expected cellular localization (Figure 3). If the localization of the signal is as expected, this antibody will pass and is considered suitable for use in IHC/ICC. We are currently using KO cell lines for our ICC validation."
• Bio-rad Antibodies, MCA1957GA Rat monoclonal andi-CD68 148455. "Rat anti mouse CD68 antibody, clone FA-11, has been used in many mouse models for the identification of CD68 in immunohistochemical studies, using both frozen and paraffin-embedded tissues (Masaki et al. 2003) and (Devey et al. 2009). This product has been reported to work in the following applications. This information is derived from testing within our laboratories, peer-reviewed publications or personal communications from the originators: Flow cytometry, Immunofluorescence, Immunohistology - Frozen, Immunohistology - Parafine, Immunoprecipitation, Western Blotting."

Antibodies received as gifts from individual labs, and not available commercially, were validated in previous studies :
• Rabbit anti-L.monocytogenes and Rabbit anti-L.innocua: Dramsi et al., 1998 and Moura et al., 2019
• Rabbit anti-Blr: Waldemarsson et al., 2006 (validated by western-blot and Elisa)

• Guinea Pig anti-LpR2: Parra-Peralbo and Culi., 2011

Homemade antibodies (Dramsi lab):
• Rabbit anti-GBS: it was validated using Dot Blot and immunofluorescence, showing that it recognised several GBS strains (NEM316, BM110, COH1, A909, 2603V/R, 515) while not reacting against closely-related bacteria (GAS, S. gallolyticus, E. faecalis, L. lactis)
• Rabbit anti-pneumoniae: this polyclonal antibody was produced against whole formaldehyde-fixed (1%) bacteria. Dot Blot and immunofluorescence showed that it recognised all S. pneumoniae strains (around 40 clinical strains) used in the Dramsi lab while not reacting against closely-related bacteria (GAS, GBS, S. gallolyticus, E. faecalis, L. lactis).

Animals and other organisms

Policy information about studies involving animals; ARRIVE guidelines recommended for reporting animal research

Laboratory animals	1. Drosophila melanogaster. A mix of males and females were used for the experiments. Larvae were all L3 stage (72h at 25°C), and adults were dissected between 4-6 days after eclosion.<br>The following strains were used: wolbachia-free w1118 (Pontier and Schweisguth, 2015), mdr65-mtd-tomato (this study), mdr65-Gal4 (BDSC 50472), UAS-mCD8-RFP (BDSC 27399 and 27400), NP6293-Gal4 ; tub-Gal80ts (Awasaki et al, 2008), UAS-shg RNAi (BDSC stock 34831), UAS-LpR1 RNAi (VDRC stock 106364), UAS-LpR2 RNAi (VDRC stocks 107597), UAS-arr RNAi (VDRC stock 4818), UAS-mgl RNAi (VDRC stock 105071), yw ; Mi(PT-GFSTF.1)LpR2MI04745-GFSTF.1(BDSC stock 60219), UAS-shits (BDSC stock 44222), UAS-shiK44A (BDSC stock 5811), yw; EGFP-Rab5 (Fabrowski et al., 2013), UAS-GFP-myc-2xFYVE; UAS-spin.myc-mRFP (BDSC stock 42716), vkg::GFP (Buszczak et al., 2007), trol::GFP (Voigt et al., 2002).<br><br>2. Mus musculus, strain CD-1, male, 8-10 week old, housed in filter-top cages under 12-h light/12-h dark cycles, with ad libitum access to food pellets and water, in a specific pathogens-free area with controlled ambient temperature and relative humidity (22 ± 2 oC/ 40-70%, respectively)
Wild animals	No
Field-collected samples	No
Ethics oversight	1. Not relevant for Drosophila, which is not subjected to lab animal regulatory rules. For transgenics use and generation we have GMO approvals from the French Government (2016-2263 and 2017-2263bis).<br><br>2. All mouse experiments in this study were carried out in the Department of Animal Models for Biomedical Research of the Hellenic Pasteur Institute in strict compliance with the European and National Law for Laboratory Animals Use (Directive 2010/63/EU and Presidential Decree 156/2013), with the FELASA recommendations for euthanasia and Guide for the Care and Use of Laboratory Animals of the National Institutes of Health. All animal work was conducted according to protocols approved by the Institutional Protocols Evaluation Committee of the Hellenic Pasteur Institute (Animal House Establishment Code: EL 25 BIO 013). License No 6317/27-11-2017 for experimentation was issued by the Greek authorities, i.e., the Veterinary Department of the Athens Prefecture. The preparation of this manuscript was done in compliance with ARRIVE (Animal Research: Reporting of In Vivo Experiments) guidelines.

Note that full information on the approval of the study protocol must also be provided in the manuscript.
